# Childhood Dysglycemia: Prevalence and Outcome in a Referral Hospital

**DOI:** 10.1371/journal.pone.0065193

**Published:** 2013-05-31

**Authors:** Emercia Sambany, Eric Pussard, Christian Rajaonarivo, Honoré Raobijaona, Hubert Barennes

**Affiliations:** 1 Institut Francophone pour la Médecine Tropicale, Vientiane, Lao PDR; 2 Génétique Moléculaire, Pharmacogénétique et Hormonologie CHU Bicêtre Kremlin Bicêtre, Paris, France; 3 Service de Pédiatrie Hôpital Befelatanana, Antananarivo, Madagascar; 4 ISPED, Centre INSERM U897-Epidemiologie-Biostatistique, Univ. Bordeaux, Bordeaux, France; 5 INSERM, ISPED, Centre INSERM U897-Epidemiologie-Biostatistique, Bordeaux, France; 6 ANRS, Phnom Penh, Cambodia; Aeras, United States of America

## Abstract

**Introduction:**

Hypoglycemia is a defining feature of severe malaria and several other infectious diseases in children but the prevalence, significance, and prognosis of abnormal blood glucose, including hyperglycemia, have rarely been addressed in severely ill children in non-malaria endemic areas.

**Methods:**

In Madagascar, consecutive children (1 month-15 years) admitted to the pediatric ward of a referral hospital, were categorized using the integrated management of childhood illness (IMCI). Samples were taken once on admission for measuring blood glucose concentration. Glycemia levels (hypoglycemia <2.2 mmol/l; low glycemia: 2.2–4.4 mmol/l; normoglycemia >4.4–8.3 mmol/l; and hyperglycemia >8.3 mmol/l) were related to the IMCI algorithm and case fatality. Factors associated with blood glucose concentration and case fatality were analysed using univariate and multivariate analysis.

**Results:**

Of 420 children, 48.1% (n = 202) were severely ill; 3.1% (n = 13) had hypoglycemia; 20.0% (n = 84) low glycemia; 65.9% (n = 277) normoglycemia; and 10.9% (n = 46) hyperglycemia. In univariate analysis, hypoglycemia and hyperglycemia both showed significant increase in the risk of death, as compared to normal blood glucose (RR: 12.2, 95% CI: 6.2–23.7 and RR: 2.5, 95% CI: 1.0–6.2, respectively). Children with low glycemia had no increased risk of death (RR: 1.2, 95% CI: 0.4–3.2) despite a poorer IMCI status on admission. After logistic regression, hypoglycemia (RR: 19.4, 95% CI: 5.0–.74.7, hepatomegaly (RR: 12.2, 95% CI: 3.3–44.9) and coma (RR: 4.8, 95% CI: 1.3–17.6) were the features on admission associated with an increased risk of death.

**Conclusions:**

Dysglycemia in non-neonates is associated with increased mortality. These findings underline the need for the use of rapid screening tests to initiate early treatment. Alternative treatments such as oral or sublingual administration of glucose should be developed in structures with limited resources.

## Introduction

Alteration of blood glucose homeostasis is common in critically ill children. In the tropics, children are particularly prone to developing hypoglycemia in a wide variety of diseases, often related to childhood malnutrition, endemic malaria, or infectious diseases [1]–[10]. A child's developing brain is more susceptible to hypoglycemia when compared with an adult's, and hypoglycemia is of particular severity in neonates and infants, or during any kind of prolonged fasting [11]. Hypoglycemia is associated with poor prognosis and neurological damage resulting in death. Well-documented associations exist between it and other indices of disease severity in malaria, such as acidosis, respiratory distress, and impaired renal function but its pathogenesis remains poorly understood [12]. In resource-poor countries hypoglycemia may be aggravated by local conditions such as: altered nutritional status; infectious diseases; delay in admittance to hospital; the use of potentially toxic herbal preparations; and the lack of diagnostic facilities [3], [13]–[14].

In critically ill non-neonate children admitted to hospital in the tropics, the prevalence of hypoglycemia has been infrequently assessed using different thresholds [2], [5], [12], [14]–[17]. The threshold of blood glucose, which defines a more critical prognosis, is still a matter of discussion with researchers suggesting a higher level than the current definition of 2.2 mmol/l [17].

Hyperglycemia in severely ill patients is also a well-known factor for increased morbidity and mortality in intensive care units [18], [19] but in the tropics data on hyperglycemia in children is scarce. In a rural Kenyan district hospital, blood glucose concentrations over 10 mmol/l were associated with a higher mortality than normoglycemia [13]. Previously, hyperglycemia in non-diabetic patients was considered to be a part of the adaptive stress response. In 2001, the control of glucose levels in critically ill patients, using strict insulin therapy, reduced overall in-hospital mortality and morbidity [20], [21].

There is a definite association between alterations of blood glucose homeostasis and the outcome for severely ill children, in admissions units. In remote and resource-poor areas, these alterations are often undetected because of the lack of useful tools for blood glucose measurements. Moreover, hypoglycemia, and even more hyperglycemia, is not easily diagnosed clinically. WHO recommends a systematic screening of severely ill children for hypoglycemia, and its treatment by administering dextrose [22]. Summative evidence suggests that the reduction of severe hyperglycemia would reduce morbidity especially from infectious diseases.

Uncertainty still persists regarding the significance, definition, and management of abnormal blood glucose in children in the tropics [16], [17].

The integrated management of childhood illness (IMCI) is a decision algorithm which aims to reduce morbidity and child mortality [23]. This tool classifies the severity of children's disease according to the level of health facilities and helps to perform a rapid triage of children requiring urgent care. IMCI efficacy in detecting hypoglycemia or abnormal blood glucose - biological data poorly evidenced by clinical signs - is unknown.

This study assessed the prevalence and outcome of abnormal blood glucose in critically ill children admitted to a university hospital in Madagascar.

## Methods

### Ethics statement

The study was performed in accordance with the Declaration of Helsinki [24]. Ethical approval was granted by the Lao Medical Ethics Committee (IFMT in Laos being the promoter of the study), and the Ministry of Health authorities in Madagascar. Parents/guardians were informed about the study in Malagasy language and given an information sheet describing the study. Children were included if their parents/guardians had given written informed consent. This was considered to be the most appropriate mode as the study was of a simple, observational design with minimal risk.

### Study setting site

The study was conducted in the admissions unit of Befelatanana University Hospital, the main pediatric institution, in Antananarivo, the capital of Madagascar, between March and June 2010. In Madagascar (19 million inhabitants, 45% under 15 years) 68% of the population lives below the poverty line of USD 1.25 per day. Per capita income was USD 950 in 2011. The illiteracy rate is higher among women (32%) than men (22.2%). Malaria is mainly endemic in coastal regions, not in the highlands [25], [26]. The under-5 mortality rate is 106 per thousand while 15% of children below the age of five years suffer from acute malnutrition and 53% from stunting [27].

IMCI was introduced in Madagascar in 1995, and is currently used in the main hospitals and 90% of districts to triage severely ill children [28], [29].

### Study site, patients and clinical procedures

The pediatric department of Befelatanana Hospital (75 beds) admits on average 3000 children per year [29]. The frequency of severe illness and general signs of danger is high in the admissions unit (56.7% and 38.2%, respectively) [29]. In-patient mortality rate dropped from 16% in 1998 (with 47.6% of deaths attributed to diarrhea) to 7% in 2009 [29]. Patients (or their parents) have to pay hospital fees and buy all necessary medical supplies (i.e. syringes and infusion sets) and prescribed medications from the pharmacy at the hospital before any treatment can begin. Incoming children are usually received at the admissions unit where a team of doctors and nurses are on duty 24 hours. The IMCI algorithm is used to triage the severity of children and determine who will be admitted to the ward, referred to peripheral units, or sent back home with a prescription [22], [29]. According to hospital guidelines, all children with general signs of danger, and all children brought in at night, are admitted to the pediatric ward, whatever their IMCI status.

### Participants

All children (1 month–15 years) admitted to the pediatric service during the investigators' duty hours were eligible into the study. Emergency cases were included only if inclusion would not cause delays in their treatment. Children with known diabetes or hemophilia, or a history of neonatal hypoglycemia, were excluded as well as children who had been previously enrolled in the study.

Investigators were on duty for 48 hours, then given a 24-hour break.

### Clinical evaluation

The medical history and examination findings were recorded in a standardized pre-tested clinical form. This form recorded demographic data, disease history, and previous treatment. Children were classified according to the algorithm of IMCI including the general danger signs: serious illness; signs of severe dehydration; signs of moderate/severe dehydration; pneumonia and nutritional status. Time and status at discharge were recorded from the hospitals forms.

Children diagnosed with hypoglycemia were treated with intravenous bolus administration of 20 mg/kg of 10% dextrose and the treatment for their baseline disease was started as early as possible. Children warranting hospitalization were sent to the pediatric ward for further treatment according to hospital guidelines.

In-hospital case fatalities were recorded prospectively for all patients admitted during the study period. Patients were discharged when the medical team considered them fully recovered. Children with aggravated status, who were discharged according to the wishes of their parents, and were not expected to survive, were recorded as additional case fatalities.

Children were weighed with 100 g precision and measured (length below 2 years, height above) with 1 mm precision. Nutritional Z-scores were calculated for children under the age of 5 years, using WHO software for anthropometrical Z scores. Malnutrition was defined as moderate or severe if one of the Z-scores was below −2 or −3 SD, respectively.

Level of consciousness was appreciated using the score routinely used in the ward: Blantyre Scale for children under 5 years, or the Glasgow Coma Score for those over.

### Definitions

Severe illness was defined according to IMCI standards: presence of any general danger signs; inability to drink or drinking poorly; vomiting; convulsions; lethargy or unconsciousness; pneumonia or severe pneumonia; severe dehydration; some dehydration; persistent diarrhea; severe persistent diarrhea; very severe febrile disease; and severe malnutrition.

Children were categorized into four groups according their blood glucose levels. The intervals of glucose blood levels were previously reported in the literature:

- Hypoglycemia: <2.2 mmol/l (<40 mg/ dl) [30].
- Low glycemia: 2.2–4.4 mmol/l (40–79 mg/ dl) [15].
- Normoglycemia: 4.4–8.3 mmol/l (80–149 mg/ dl).
- Hyperglycemia: over 8.3 mmol/l (≥150 mg/ dl) [17], [19].

Abnormal blood glucose, or dysglycemia, was defined as any of the 3 categories which differ from normoglycemia.

### Laboratory measurements

As soon as it was possible after arrival, and after informed consent, 0. 6 µL of blood was collected by investigators through a finger prick to measure the blood glucose concentration (Accu-Chek® Performa glucometers from ROCHE Laboratories, with a sensitivity limit of 1 mmol/l according to the manufacturer). After every twenty-fifth measurement, a quality control by Accu-Chek® was performed. Blood glucose concentrations were recorded in mmol/l (conversion to mg/dl by multiplying by a factor of 18).

### Outcome

The main outcome was the prevalence of dysglycemia. Secondary outcomes were: IMCI status; deaths; proportion of deaths within 24 hours; and time before discharge.

### Statistical analysis

Data was entered in EpiData freeware. All records were cross-checked with the original data sheets. Analyses was carried out with STATA, Version 8 (Stata Corporation, College Station, TX, USA) and eventually re-checked with free software Open Epi software (http://www.openepi.com/Downloads/Downloads.htm).

Chi squared or Fisher's exact tests were used to compare categorical variables as appropriate. Data distribution was graphically evaluated using the kemel density estimate and eventually tested with the Skewness and Kurtosis test and the Shapiro-Wilk test for normality. Kruskal-Wallis test was used for non-normally distributed variables.

Relative risks (RR) were calculated with Taylor series 95% confidence intervals. We considered p <0.05 as statistically significant.

Children were classified into four glycemia categories (hypoglycemia, low glycemia, normoglycemia and hyperglycemia) and into 2 IMCI categories (severe, non-severe). All dysglycemic groups were compared to normoglycemic children. Associations between admissions glycemia and case fatality (died in hospital or taken home critically ill, not expected to survive) were investigated using univariate analysis. Mortality (died in hospital or taken home critically ill, not expected to survive) was evaluated in univariate analysis according to: child's gender; age (less or more than 3 years); duration of hospitalization; IMCI severity of illness; associated severe illness; dysglycemic status; residential area; mother's age; education; occupation; and daily expense on food (used as an estimated proxy for poverty). Variables with a degree of significance below 0.2 for mortality were then fitted into a multivariate logistic regression model with backward step-by-step analyses using odd ratios (Stata 8) to evaluate the factors associated with child in-patient mortality.

We have attempted to report the study according to the STROBE guidelines [31].

## Results

**Figure 1:**
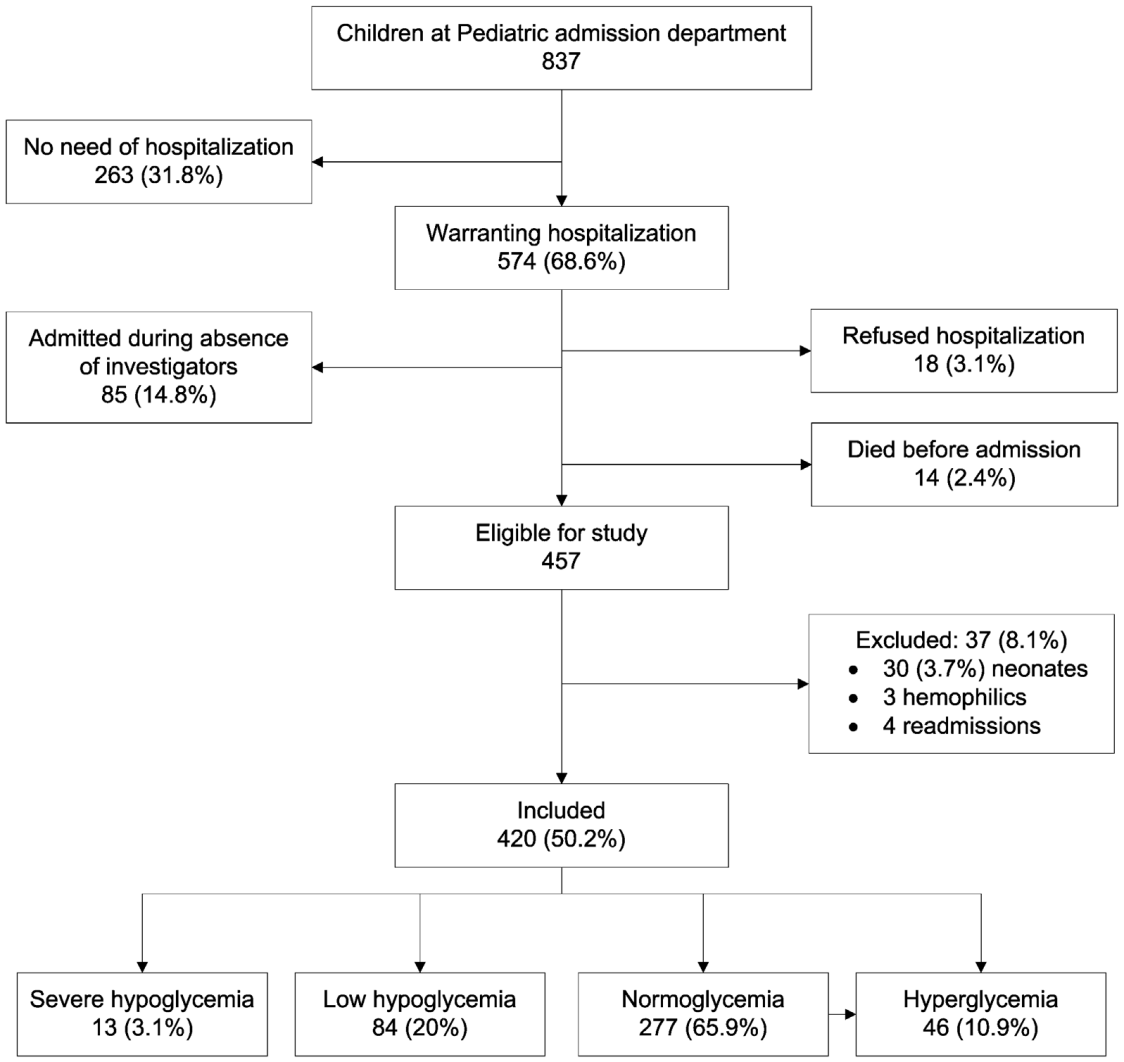
Enrolment flow chart.

A total of 837 children attended the emergency unit from March to July 2010 and 574 (68.6%) were admitted to the pediatric ward. Of them, 18 (3.1%) refused to be hospitalized, and 14 (2.4%) died on arrival before any treatment could be performed. A total of 457 were admitted when investigators were on duty. Thirty-seven children (8.1%) were excluded leaving 420 children for the analyses (chart flow of patients Figure 1).

The socio-demographic characteristics of the 420 children, according to their blood glucose at baseline are shown in Table 1. Their medical characteristics according to IMCI classification are summarized in Table 2.

**Table 1:** Socio-demographic characteristics of children on admission in Pediatrics.

Characteristics	Hypoglycemia	Low glycemia	Normal glycemia	Hyper glycemia	p	Total
	13 (3.1%)	84 (20%)	277 (65.9%)	46 (10.9%)		420
Glycemia	1.9 [1.8–2.1]	3.66 [3.5–3.8]	5.8 [5.6–5.9]	9.2 [8.8–9.6]	<0.000	5.4 [5.2–5.6]
Females	6 (46.2)	35 (41.7)	129 (46.6)	25 (54.4)	0.6	195 (46.4)
Age (months)	5.5 [1.7–9.1]	22.1 [15–29.1]	27.7 [22.8–32.7]	36.2 [21.1–51.3]	0.2	26.8 [22.9–30.7]
Weight (Kg)	7.2 [4.4–10]	10.2 [8.9–11.5]	11.9 [10.9–12.8]	12.7 [9.9–15.5]	<0.01	11.5 [10.7–12.2]
Live in main city	10 (76.9)	66 (78.6)	221 (79.8)	31 (67.4)	0.3	328 (78)
Duration of illness (days)	3.6 [3.3–3.9]	3.1 [2.6–3.7]	3.8 [3.3–4.3]	3.3 [2.5–4.5]	0.4	3.6 [3.3–3.9]
Over 2 hours fasting	10 (76.9)	50 (59.5)	121 (43.7)	25 (54.4)	<0.01	206 (49.1)
Day time admission (6am 6pm)	9 (69.2)	61 (72.6)	217 (78.3)	33 (71.7)	0.5	320 (76.2)
Taking anti-malarial in the last 24 hours	2 (15.4)	4 (4.8)	21 (7.6)	4 (8.7)	0.5	31 (7.4)
Never vaccinated	1 (7.7)	5 (5.9)	10 (3.6)	4 (8.7)	0.4	20 (4.8)

Mean and [95% confidence interval (95% CI)].

**Table 2:** Medical characteristics of children according to IMCI algorithm.

Characteristics	Hypoglycemia	Low glycemia	Normal glycemia	Hyperglycemia	p	Total
	13 (3.1%)	84 (20%)	277 (65.9%)	46 (10.9%)		420
**General danger signs**						
Unable to drink or to breastfeed	5 (38.5)	7 (8.3)	11 (3.9)	3 (6.5)	<0.000	26 (6.2)
Vomits everything	7 (53.9)	34 (40.5)	65 (23.5)	8 (17.4)	<0.001	114 (27.1)
Lethargy or unconsciousness	11 (84.6)	17 (20.2)	44 (15.9)	11 (23.9)	<0.000	83 (19.8)
Convulsions	1 (7.7)	13 (15.5)	41 (14.8)	11 (23.9)	0.4	66 (15.7)
**Severe disease**	11 (84.6)	49 (58.3)	121 (43.7)	21 (45.7)	<0.005	202 (48.1)
Severe pneumonia	2 (15.4)	10 (11.9)	48 (17.3)	7 (15.2)	0.6	67 (15.9)
Pneumonia	3 (23.1)	23 (27.4)	105 (37.9)	19 (41.3)	0.12	150 (35.7)
Severe dehydration (with diarrhea)	7 (53.9)	9 (10.7)	13 (4.7)	4 (8.7)	<0.000	33 (7.9)
Sunken eyes	7 (53.9)	25 (29.8)	48 (17.3)	6 (13)	<0.001	86 (20.5)
Unable to drink or drinking poorly	5 (38.5)	7 (8.3)	11 (3.9)	3 (6.5)	<0.0001	26 (6.2)
Skin pinch goes back very slowly	6 (46.2)	23 (27.4)	47 (16.9)	6 (13)	<0.009	82 (19.5)
Some dehydration (with diarrhea)	0	18 (21.4)	44 (15.9)	6 (13)	0.2	68 (16.2)
Restless, irritable	0	15 (17.9)	15 (5.4)	2 (4.4)	<0.001	32 (7.6)
Thirsty	3 (23.1)	5 (5.9)	18 (6.5)	1 (2.2)	0.06	27 (6.4)
Persistent diarrhea	0	2 (2.4)	0	0	0.04	2 (0.5)
Very severe febrile disease	4 (30.8)	20 (23.8)	44 (15.9)	13 (28.3)	1.8	81 (19.3)
Severe malnutrition*	12 (92.3)	64 (76.2)	205 (74)	33 (71.7)	0.5	314 (74.8)

IMCI: integrated management of childhood illness.

* Weight for height, or height for age, or weight for age ≤3 Standard Deviation (WHO 2006) among children less than 59 months.

14 had kwashiorkor (1 hypo, 2 low glycemia, 10 normoglycemia, 1 hyperglycemia, 1 hyperglycemia had marastic-kwashiorkor).

Of 420 children, 13 (3.1%) had hypoglycemia, 84 (20%) low glycemia, 277 (65.9%) normoglycemia, and 46 (10.9%) hyperglycemia. The overall distribution of blood glucose is skewed, with a range of 1.6–18.3 mmol/l (Shapiro-Wilk test p<0.001) and 143 (34.1%) had dysglycemia.

Children with hypoglycemia tended to be younger and weighed less than those with normoglycemia, though this did not reach significance. Hypoglycemia was also associated with more frequent fasting (p = 0.01). Thirty-one (7.4%) children had received some form of anti-malaria drugs before admission, but none of these treatments were associated with the level of glycemia. Only 2 children had imported cases of severe malaria. Mothers' occupations were: shopkeepers 192 (45.7%), unemployed 101 (24.0%), civil servants 50 (11.8%), unskilled workers 41 (9.76%), workers 20 (4.8%), farmers 16 (3.8%). The distribution of mothers' occupations was not associated with the level of blood glucose on admission.

Of 420 children, 48.1% (n = 202) were severely ill, and 289 (68.8%) had at least one sign of general danger.

Children with hypoglycemia were more frequently unconscious or vomiting. More often than others, they had a severe disease, severe dehydration, or severe malnutrition.

Convulsions and very febrile diseases were more frequent among children with hyperglycemia than normoglycemia (OR: 1.8, 95% CI: 0.7–4.0 and OR: 2.0, 95% CI: 0.9–4.4, respectively) without reaching significance. Very febrile diseases tended also to be more frequent among children with hypoglycemia than normoglycemia (OR: 3.0, 95% CI: 0.6–12.4) without reaching significance.

Irritability was more frequent among children with low glycemia. The incidence of severe pneumonia was similar within the 4 groups. Of 420 children, 15 (3.6%) had hepatomegaly though none in the hypo group. The distribution was similar in the dysglycemic groups (3.3%, 4.7%, and 4.3% in normal, low and hyperglycemia groups, respectively).

Of 420 children, 7 (1.7%) had cardiac failure. The frequency of hepatomegaly (n = 15, 3.5%) was higher in children with cardiac failure (n = 4; 57.1%) than in those without (n = 11; 2.7%) (p<0.0001).

Median baseline blood glucose between deaths and survivors were 4.9 mmol/dl, IQR: 2.1–7.4 and 5.2 mmol/dl, IQR: 4.4–7.0, respectively.

Case fatality rates of children according to glucose blood levels are described in Table 3. The overall mortality was 7.8% (95% CI: 5.4–10.8). Mortality was especially high 19/33 (57.6%) during the first 24 hours of admission.

**Table 3:** Case fatality according to blood glucose concentration of 420 admitted to Pediatrics.

Evolutionary aspect	Hypoglycemia	Low glycemia	Normal glycemia	Hyperglycemia	p	Total
	13 (3.10%)	84 (20%)	277 (65.95%)	46 (10.95%)		420
Age at death (month)	6.4 [1.1–11.7]	6.4 [0–16.5]	28.3 [0–59.8]	28.3 [0–70.7]	0.5	19.7 [5.5–33.8]
Death*	8 (61.5)	5 (5.9)	14 (5.1)	6 (13.0.)	<0.000	33 (7.8)
Death within 24H	5 (38.5)	4 (4.8)	5 (1.8)	5 (10.9)	<0.000	19 (4.5)
Time to deaths (hrs)	1.7 [0–2.9]	1 [0–2.6]	6.1 [0–13.3]	1 [0–2.3]	0.1	3.3 [0–6.4]
Transferred**	1 (7.7)	12 (14.3)	36 (13.0)	2 (4.4)	0.3	51 (12.1)
Duration of hospitalization in survivors	7.2[0–3.0]	5.2 [3.6–6.7]	4.2 [3.7–4.7]	4.1 [2.9–5.1]	0.9	4.4 [3.9–4.9]

Mean and [95% confidence interval (95% CI)].

* Died during hospitalization or left critically ill and expected to died.

** Children were transferred to nutrition rehabilitation centre after improvement of acute status, 7 children left the hospital without notice, their outcome is unknown.

Children with hypoglycemia or hyperglycemia had the highest risk of death (RR: 12.2 95% CI: 6.2–23.7 and RR: 2.5, 95% CI: 1.0–6.2, respectively) (Table 4). Compared to children with normal glycemia, children with low glycemia had no increased risk of death (RR: 1.2, 95% CI: 0.4–3.2) despite a more severe disease at admission.

**Table 4:** Baseline glycemia and children features and in-hospital mortality (univariate analysis)*.

Outcomes	Mortality n (%)	RR [95% CI]	p
**Baseline glycemia**			
Euglycemia	14/277 (5.0)	1	
Hypoglycemia	8/13 (61.5)	12.18 [6.24–23.73]	<0.000
Low glycemia	5/84 (5.9)	1.17 [0.43–3.17]	0.92
Hyperglycemia	6/46 (13.0)	2.52 [1.02–6.24]	0.05
Dysglycemia	19/143 (13.2)	2.62 [1.35–5.087]	0.003
**Socio-characteristics**			
Male	13/225 (5.7)		
Female	20/195 (10.2)	1.775 [0.90–3.47]	0.06
≥3 years	3/104 (2.8)	1	
<3 years	30/316 (9.4)	0.11[0.03–0.354]	<0.000
Rural residential area	12/92 (13.0)	1	
Urban residential area	21/328 (6.4)	2.03[1.04–3.98]	0.03
**Education (Mother)**			
Illiterate	0/2 (0)	–	
Primary	15/117 (12.8)	3.141 [1.07–9.15]	
Secondary	14/203 (6.9)	1.69 [0.57–4.99]	
High school	4/98 (4.0)	1	
			
Not Poor	16/282 (5.6)	1	
Poor	17/138 (12.3)	2.171 [1.13–4.16]	0.03
			
Illness <2 days	2/160 (1.25)	1	
Illness ≥2 days	31/260 (9.0)	0.1048 [0.02–0.43]	<0.000
			
Non severe disease	6/169 (2.7)	1	
Severe disease	27/251 (10.7)	3.03 [1.27–7.18]	0.004
Conscious	25/385 (6.4)	1	
Coma	8/35 (22.8)	3.52 [1.71–7.21]	0.003
No hepatomegaly	28/405 (6.9)	1	0.004
Hepatomegaly	5/15 (33.3)	4.8 [2.16–10.73]	

* Only significant factors, Fischer's exact test when expected value of cell value is <5. CI: confidence interval.

Children classified as IMCI severe had an increased risk of death (RR: 3.0, 95% CI: 1.2–7.2). The distribution of mothers' occupations was not associated to the mortality: shop keepers 16/192 (8.3%), unemployed 6/101 (5.9%), civil servants 2/50 (4.0%), unskilled workers 4/41 (9.8%), workers 2/20 (10.0%), farmers 1/16 (6.3%).

IMCI had a high sensitivity but low specificity in detecting hypoglycemia (84.6% and 31%, respectively) and low sensitivity and specificity for dysglycemia (57% and 56%, respectively).

The univariate analyses of factors associated with deaths is shown in Table 4. In univariate analyses severe IMCI, coma, hypoglycemia and hyperglycemia and a longer duration of the disease (over 2 days) increased the risk of death. In multivariate analyses, hypoglycemia (RR: 19.4, 95% CI: 5.0–.74.7, hepatomegaly (RR: 12.2, 95% CI: 3.3–44.9,) and coma (RR: 3.4, 95% CI: 1.7–7.0) were the features associated with the increased risk of death.

## Discussion

These findings show that abnormal blood glucose concentration is common in severely ill children in tropical settings [143/420 (34.1%)], and is associated with the increased risk of death (19/143 = 13.3% versus normoglycemic 14/277 = 5.1%, p = 0.003), even in areas without endemic malaria [10], [13], [17], [31]. Hypoglycemia was associated with severe IMCI status. It was less frequent (3.1%) in Madagascar than previously reported in other tropical settings. It was associated with an increased case fatality (61.5%) despite the implementation of protocols for blood glucose measurement and appropriate treatment in the admissions unit. The majority of the 420 children admitted had a severe IMCI status and 14 children died on arrival at the admissions triage. Nineteen of the 33 deaths (57%) after inclusion took place during the first 24 hours. These results showed that children who had reached the emergency unit in a tropical setting were already critically ill [29].

Low glycemia was the most common glycemic dysregulation (20%) but was not associated with mortality, despite a poorer IMCI status than children with normal glycemia [17]. On the other hand, both hypoglycemia and hyperglycemia carried an increased mortality rate [13]. These results support the recommendation of glycemia determination in the management of sick children [22]. IMCI-classified severe disease was associated with poor prognosis and related to hypoglycemia. However the IMCI algorithm was a tool of low specificity for detecting dysglycemia.

### Hypoglycemia prevalence, detection and treatment

The overall prevalence of hypoglycemia in this non-malaria setting of Madagascar was lower than in some other studies regarding children with malaria [8], [11]–[13], [14], [32] but was similar to that previously observed by us in a high malaria-endemic area in Mali (3%) [3].

Though it is difficult to compare health facilities between different countries, endemic malaria enhanced the risk of hypoglycemia and may explain, at least partially, the differences observed [10], [13]. During malaria, parasitized erythrocytes consume 30 to 75 times the quantity of glucose that non-infected cells require and endogenous production of glucose is unbalanced by its over utilization [11].

Hyperinsulinemia after quinine treatment is a well-established iatrogenic cause linked to increased mortality [12], [33], [34], [35].

In addition, hypoglycemia in children is independently associated with poor outcome in the tropics. It was reported during malaria episodes [14], infections [11], pneumonia [5], diarrhea [11], [36], malnutrition [2], and intoxication [1], [37]. It is aggravated by fasting and vomiting [9].

Time between the last meal and unconsciousness was associated with hypoglycemia. Prolonged duration of illness and the delay of referral before admission increased the fasting period. Younger children and neonates are particularly susceptible to hypoglycemia, both in tropical and western countries [38]. Compared to adults, children have a limited tolerance to fasting because glycogen storage capability is less and therefore they are able to maintain a normal plasma glucose level for a fasting period of 12 hours only [39]. Anorexia induced by the disease, and traditional habits shorten the tolerance to fasting.

The etiology of hypoglycemia is not completely understood and is likely to be multifactorial. During acute infectious diseases, hypoglycemia may result in reduced calorie intake and stress-induced cytokines. Glucose metabolism is similar in children and adults in terms of glycogenolysis and glycolysis. However, carbohydrate stocks are limited in children and glucose storage is depleted by starvation. Parasite utilization or cytokine-induced impairment of gluconeogenesis has been implicated in the mechanism of hypoglycemia. During critical illnesses, the physiological responses induced by hypoglycemia may be frequently impaired. Prolonged hypoglycemia may induce biological toxicities, such as increased systemic inflammatory response, cerebral vasodilation, and neuroglycopenia, resulting in neuronal excitotoxicity and cellular damage.

Estimating hypoglycemia at the bedside is particularly difficult due to the lack of specificity of the clinical signs such as the usual vegetative symptoms observed in healthy children [13]. Critically ill or comatose children do not develop specific symptoms despite hypoglycemia levels lower than 1.2 mmol/l. Moreover, hypoglycemia also remains undetected biologically because of limited laboratory resources. Rapid bedside glucose tests are seldom available but should be considered a crucial first-aid element, in the management of a sick child [40]. Despite concerns at the underestimation in cases of moderate to severe anemia and overestimation of hypoglycemia for glucose levels below 3 mmol/l, [30] the usefulness of bedside tests is essential in areas with limited resources and in emergency units. The glucometer was found to be easy to use by health workers, and sensitive enough for blood sugar determination in emergency cases for confirming the diagnosis [41].

Early correction of hypoglycemia is an important therapeutic measure as it is associated with increased mortality though more research is needed to show evidence that systematically preventing it could reduce child mortality in the tropics [12], [17].

WHO guidelines now recommend intravenous treatment of ‘symptomatic hypoglycemia’ using 10% glucose, with continued monitoring and feeding or continued glucose infusion thereafter [22]. However, IV therapy is only feasible where trained personnel and essential supplies are available. Longer delay in initiating resuscitative measures is a contributory factor to early death and should be addressed in tropical pediatric health facilities. In our study, more than 50% of the deaths occurred during the first 24 hours after admission and 14 others died before being able to enter the pediatric ward. This high mortality could be explained by the frequent non-availability of emergency medications in the pediatric wards, or by excessive delay in providing care at the referral hospital [29]. In many hospitals, as here, parents have to buy all necessary medical equipment and prescribed medications from a pharmacy, losing precious time in the race for the survival of their children. Attempts to keep emergency medications in the facility are regularly hindered by financial constraints.

In resource-constrained settings where administering intravenous dextrose is delayed or impossible, there is increasing evidence that sub-lingual administration of sugar could be an immediate and effective ‘first-aid’ measure [3], [15]–[16].

### Low glycemia prevalence and prognosis

Low glycemia was the most common blood glucose dysregulation (20%) and was associated with a poorer IMCI status than in children with normal blood glucose. Contrary to the findings in our previous study in Mali, low glycemia was not associated with increased mortality suggesting the additional role of malaria as a factor of mortality there [17]. These children were older than those with hypoglycemia, suggesting a better tolerance to the illness. Furthermore, the children who died in this low glycemia group were as young as those in the hypoglycemia group. The frequency of vomiting and the duration of the fasting period were more important than in the normoglycemic group, contributing to the decrease of blood glucose concentration. This suggests recommending the continued feeding of severely ill children. This data also suggests that low glycemia should be assessed further in young children as a pejorative factor associated with their deaths.

### Hyperglycemia prevalence, prognosis, significance and treatment

There are no definite criteria for diagnosing hyperglycemia at admissions among children without diabetes mellitus. However a strong association was demonstrated between the risk of dying and the likelihood of having a persistent glucose blood level higher than 150 mg/dl in critically ill children [42].

In our study, hyperglycemia, a common dysglycemia (11%) carried an increased risk of death (RR: 2.2, 95% CI: 1–4.7). It was diversely evaluated in previous studies. In Mali, hyperglycemia was associated with fewer deaths and considered a potentially protective mechanism in children with severe malaria [17]. However, hyperglycemia was more often associated as a pejorative aggravating factor in another report [13] and in emergency units [43].

As previously described in Mali, hyperglycemia was associated with more frequent seizures. About 15.7% of our patients with convulsions had a blood glucose concentration greater than 8.3 mmol/l at admission. In an Italian study, 12.9% of children with febrile convulsions had stress hyperglycemia [19].

In an American study in a community pediatric hospital, hyperglycemia was associated with a greater need for intensive care and ICU monitoring but not with increased in-hospital mortality [44]. In a similar study, the frequency of hyperglycemia was evaluated at 14.3% for children admitted to intensive care centers without any association between hyperglycemia and mortality [45].

On the contrary, our results in Madagascar are in agreement with the Kenya study which suggests that the difference in mortality in ICU settings and their centre at Kilifi is based on the lack of ICU support [17], [19], [46]. A higher mortality rate was observed among hyperglycemic (13.0%) than in normoglycemic (5.0%) children, suggesting a greater severity state. The role and proper treatment of hyperglycemia in the tropics should be addressed further.

While it is well-known that hyperglycemia is a possible component of the neuroendocrine response to stress, it is not clear how different stimuli may trigger it. Stress response commonly includes elevations in plasma concentrations of hyperglycemic hormones like glucocorticoids, catecholamines, glucagon and growth hormones resulting in alterations to the metabolism of glucose and other energy substrates. Insulin concentrations may be inappropriately low for serum glucose concentration, or insulin may have diminished receptor responsiveness in seriously stressed patients. Many alterations in carbohydrate metabolism have been proposed including increased gluconeogenesis, depressed glycogenesis, glucose intolerance and insulin resistance consecutive to decreased glucose uptake in skeletal muscle. It is not always clear when an altered metabolic or hormonal state is an appropriate response to stress, or represents a general failure of the body.

The cellular glucose overload induces excessive glycolysis and oxidative phosphorylation with increased production of reactive oxygen species. These highly reactive species lead to increased apoptosis, and consequently, cellular death and organ system failure.

Clinical conditions such as traumatic injuries, febrile seizures and elevated body temperature (>39°C) are often associated with hyperglycemia. It has been suggested that cytokines, commonly increased during febrile diseases like interleukin-1 or tumor necrosis factor, may inhibit insulin release and increase the secretion of cortisol, further explaining hyperglycemia. In association with fever and seizures, hyperglycemia was found to be three times more frequent than that observed in other febrile conditions.

Until recently, hyperglycemia was not routinely controlled in ICUs, except among patients with known diabetes mellitus. Recent investigations have demonstrated that glycemic management with insulin therapy reduces morbidity and mortality in those critically ill. The appreciable frequency (11%) of hyperglycemic children at admission, and the association with an increased risk of death raise concerns regarding the use of such presumptive treatment. Further studies are needed for a better understanding of hyperglycemic outcomes and the necessity to initiate insulin treatment in severely ill hyperglycemic children admitted to pediatric emergency wards in the tropics.

The very short period between admission and death is a common proxy of tropical pediatric wards where, unfortunately, children are taken too late and in conditions beyond treatment. The fact that 18 children died before being admitted, emphasize this problem. Part of the solution lies in early diagnosis, triage and treatment of severely ill children. This is dependent on improved diagnostic tools such as IMCI classification and rapid tests, improved quality and accessibility to primary care, and the efficacy of the referral system [47].

### Limitations

This study presents some limitations. It was performed in field conditions within a busy pediatric ward of a university hospital. Also, children admitted outside the duty hours of the investigators could not be included. However, using periods of 48 consecutive hours probably decreased a potential selection bias, and results regarding the severity of patients are very similar to those presented the previous year. The 14 children who died on arrival were not assessed. This may have underestimated the rate of hypoglycemia, and partially explains the differences with other studies.

Comparisons with other studies are difficult due to the different thresholds used for hypoglycemia or hyperglycemia. Additionally, health facilities and access to health care differ widely within the areas.

### Conclusions

Hypoglycemia and hyperglycemia are associated with a high risk of mortality for children admitted to general pediatric hospitals in non-malaria areas. The pejorative prognosis of low glycemia was observed only in young children. These findings underline the need for the use of rapid screening tests to initiate early treatment. Alternative treatments such as oral or sublingual administration of glucose should be developed in structures with limited resources.
